# Autologous Conditioned Plasma (ACP) and Osteoarthritis of the Knee: A Review of Current Clinical Evidence

**DOI:** 10.7759/cureus.52693

**Published:** 2024-01-21

**Authors:** Ashim Gupta, Vijay Jain

**Affiliations:** 1 Regenerative Medicine, Future Biologics, Lawrenceville, USA; 2 Regenerative Medicine, BioIntegrate, Lawrenceville, USA; 3 Orthopaedics, South Texas Orthopaedic Research Institute, Laredo, USA; 4 Orthopaedics and Regenerative Medicine, Regenerative Orthopaedics, Noida, IND; 5 Orthopaedics, Atal Bihari Vajpayee Institute of Medical Sciences, Dr Ram Manohar Lohia Hospital, New Delhi, IND

**Keywords:** lp prp, leukocyte-poor platelet-rich plasma, prp, platelet-rich plasma, acp max, acp, autologous conditioned plasma, orthobiologics, gonarthrosis, knee osteoarthritis

## Abstract

The primary objective of this study is to record the clinical outcomes of autologous conditioned plasma (ACP) for the treatment of knee osteoarthritis (OA) based on published literature. Multiple databases (PubMed, Embase, Web of Science and Scopus) were searched using terms for “knee OA” and the intervention “ACP” for articles published in English to January 4, 2024. All clinical studies using ACP for knee OA were included. Studies not utilizing ACP alone, i.e. used as an adjunct with other modalities or not focusing on the management of knee OA, were excluded. Five studies, three randomized controlled trials (RCTs) and two real-world post-market studies conducted in a clinical practice met the inclusion/exclusion criteria and were included in this study. All studies demonstrated statistically significant improvements in various patient-reported outcome measures (PROMs), however the studies performed in the clinical practice reported non-accomplishment of minimally clinically important difference (MCID). The results demonstrated the potential of ACP for management of knee OA, however the MCID was not achieved in real-world clinical settings. Thus, more adequately powered RCTs with longer follow-up as well as real-world post-market studies are warranted to establish long-term efficacy and justify routine clinical use, respectively, of ACP in patients suffering with knee OA.

## Introduction and background

Osteoarthritis (OA) is the leading joint complaint impacting millions of people globally [1]. OA is a degenerative joint condition mostly concerning large weight-bearing joints, including knees [1]. Its aetiology comprises of inflammation of synovial tissue and deterioration of articular cartilage, resulting in pain, diminished function and affected overall quality of life (QoL) [2]. Knee OA is conservatively managed via non-pharmacological modalities such as weight reduction, activity alteration, and physical therapy; pharmacological substances such as non-steroidal anti-inflammatory drugs (NSAIDs) and opioids, and intraarticular administration of corticosteroids and viscosupplementation; minimally invasive procedures such as radiofrequency ablation of genicular nerve and peripheral nerve stimulators; and surgical interventions, in advanced stages or after conventional therapies have been unproductive [1-3]. These above-mentioned treatment modalities have shortcomings and side effects, constantly intending to decrease pain instead of targeting the causal pathophysiology [1-3].

Lately, there has been a notable interest in the use of orthobiologics, including autologous peripheral blood-derived orthobiologics (APBO) for musculoskeletal regenerative medicine [4]. Various APBO include platelet-rich plasma (PRP), autologous conditioned plasma (ACP), platelet lysate (PL), autologous conditioned serum (ACS), Gold-induced cytokine (GOLDIC), plasma-rich in growth factors (PRGF), growth factor concentrate (GFC), autologous protein solution (APS), platelet-rich fibrin (PRF) and hyperacute serum (HS). PRP is the most widely used APBO, nonetheless its efficacy remains contentious, attributed to absence of standardized preparation protocol, inter- and intra-patient variables, etc. [4]. ACP (Arthrex, Naples, FL, USA), a commercially available single-spin leukocyte-poor PRP, formulated per manufacturer's instructions, is commonly used in clinical practice for regenerative medicine applications, including for knee OA treatment [5]. To date, only a limited number of studies have investigated the efficacy of ACP for the treatment of knee OA. The primary objective of this study is to record the clinical outcomes of ACP for the treatment of knee OA. The secondary objective is to document the ongoing clinical studies registered on different trial protocol repositories related to ACP for the management of knee OA.

## Review

Search criteria

**Figure 1:**
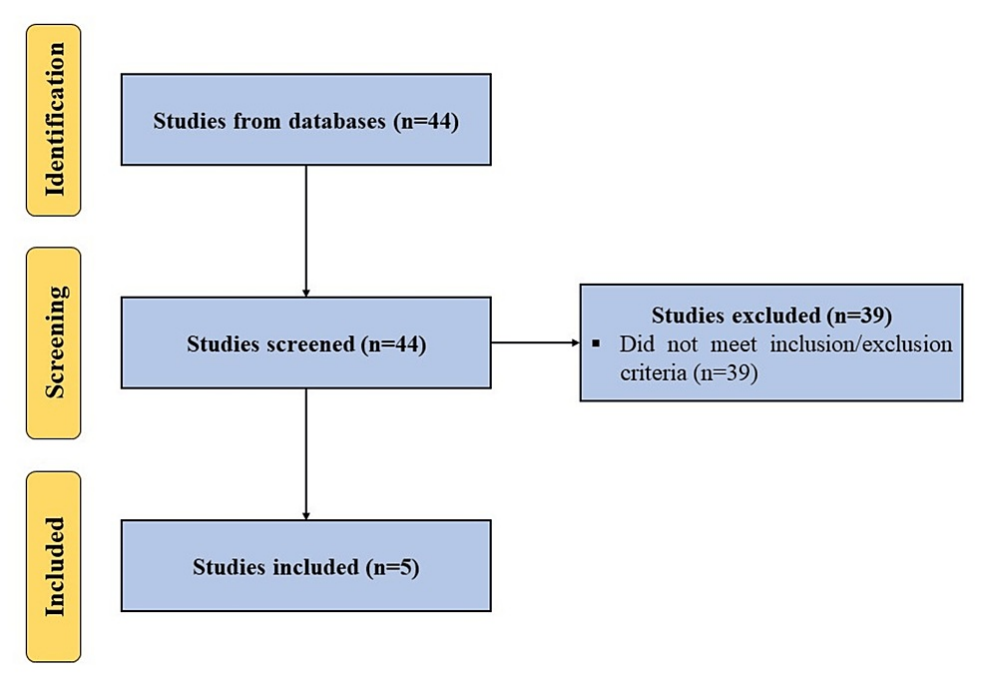
A PRISMA flow diagram outlining the record identification and selection process. PRISMA: Preferred Reporting Items for Systematic Reviews and Meta-Analyses

A search was performed using terms, (‘autologous conditioned plasma’ OR ‘ACP’ OR ‘leukocyte-poor platelet-rich plasma’ OR ‘LP PRP’) AND (‘knee osteoarthritis’), in databases including PubMed, Embase, Web of Science and Scopus for articles published in English to January 4, 2024, while adhering to Preferred Reporting Items for Systematic Reviews and Meta-Analysis (PRISMA) guidelines. All clinical studies using ACP for knee OA were included. Studies not utilizing ACP alone or not focusing on the management of knee OA were excluded (Figure 1). In addition, we searched ClinicalTrials.gov, Clinical Trials Registry - India (CTRI), and Chinese Clinical Trial Register (ChiCTR) using the above-mentioned search terms to identify registered ongoing studies on the use of ACP for the management of knee OA.

Results

Clinical Studies

Korpershoek et al. [6] in a prospective case series investigated the effect of ACP in patients with knee OA. ACP was prepared per instructions from the manufacturer (Arthrex). Three weekly intra-articular injections were administered. The patient-reported outcome measures (PROMs) were collected at baseline and at three, six, and 12 months follow-up, including Knee Injury and Osteoarthritis Outcome Score (KOOS), EuroQol 5 Dimensions 5 Levels (EQ5D-5L), and numeric pain rating scale (NPRS). Two hundred sixty patients (307 knees) were included in this study. Statistically significant improvements in the KOOS and NPRS were reported at all follow-up time points compared to the baseline, however no significant improvement was noted for the EQ5D-5L. Interestingly, despite the significant improvement in the KOOS score, most of the patients did not achieve improvements above the minimally clinically important difference (MCID). A small number of patients who achieved MCID underwent repeat cycle of three weekly ACP injections. The subgroup analysis showed better improvement in older patients and bilateral treatment led to poorer outcomes. The main limitation of this study is the lack of a placebo group or active controls. In summary, MCID was not reached post-treatment with ACP in majority of the knee OA patients, and thus, was not recommended as a routine therapy in clinical settings.

Korpershoek et al. [7] in a prospective case series investigated the effectiveness of intra-articular administration of ACP in knee OA patients. ACP was prepared per instructions from the manufacturer. One hundred forty patients (158 knees) were treated with three ACP injections. The PROMs assessed included KOOS, NPRS and EQ5D at baseline, and at three, six, and 12 months follow-up. Statistically significant improvement in the KOOS was reported at all follow-up time points compared to the baseline, however no significant improvements were noted for the NPRS and EQ5D. Similar to the aforementioned study, despite statistically significant improvements in the KOOS score, the MCID was not attained in the majority of the patients. This study has several limitations including lack of control group(s), variability in dosing frequency, and loss of patients during follow-up. In summary, intraarticular administration of ACP did not lead to MCID in knee OA patients, and thus, was not recommended as a routine therapy in clinical settings.

Cole et al. [8] in a prospective, double-blind, randomized controlled trial, compared the effects of PRP to hyaluronic acid (HA) in mild to moderate knee OA patients. One hundred eleven patients were enrolled in this study and randomized 1:1 in the PRP and HA groups. Three weekly injections of PRP or HA were administered. ACP was used as a leukocyte-poor PRP formulation and formulated per manufacturer’s instructions. Patients were evaluated at baseline, treatment weeks two and three, and at six, 12, 24 and 52 weeks follow-up. The primary outcome measure was the Western Ontario and McMaster Universities Osteoarthritis Index (WOMAC) pain subscale. The secondary outcome measures included the International Knee Documentation Committee (IKDC), visual analog scale (VAS), and Lysholm knee score. Statistically significant improvements in the IKDC and VAS scores were observed at 24 and 52 weeks follow-up compared to the baseline. However, there was a decline in improvements in both IKDC and VAS scores for both PRP and HA groups after 24 weeks follow-up. In addition, no statistically significant differences were observed for the primary outcome measure, WOMAC pain subscale, or other secondary outcome measures at any follow-up time-points compared to the baseline. In summary, despite demonstrating no improvement in the WOMAC pain subscale, administration of PRP can lead to improvement in symptoms of knee OA patients.

Smith et al. [9] in a prospective, randomized, double-blind study investigated the safety and efficacy of ACP for the treatment of knee OA. One hundred fourteen patients were screened and 30 were included in the study. The included patients were randomized 1:1 into the ACP and saline groups and received three weekly intraarticular injections. ACP was formulated per manufacturer’s instructions. The primary outcome measure was WOMAC scale, evaluated at baseline and at one week, two weeks, and two, three, six, and 12 months follow-up. No adverse events associated with administration of ACP were reported throughout the duration of the study. Statistically significant improvements in overall WOMAC were observed beginning two weeks till 12 months follow-up for the ACP group compared to the baseline as well as the saline group. The main shortcomings of this study are small sample size and lack of active comparator. In summary, administration of ACP is safe and potentially efficacious in knee OA patients.

Cerza et al. [10] in a randomized controlled trial (RCT) evaluated the efficacy of PRP with HA in gonarthrosis patients. One hundred twenty patients were enrolled in this study. Patients were consecutively randomized to either receive four intraarticular injections of ACP or HA. The primary outcome measure was WOMAC scale, assessed at baseline and at four, 12 and 24 weeks post first injection. Statistically significant improvements in the WOMAC score were observed in the ACP group compared to both the baseline as well as the HA group at 24 weeks follow-up. The main limitations of the study include lack of blinding, small cohort size and short follow-up duration. In summary, administration of ACP led to better clinical outcomes compared to HA, and thus has potential to manage gonarthrosis patients.

Ongoing Clinical Studies

As of January 4, 2024, there are three clinical trials registered on ClinicalTrials.gov, CTRI, or ChiCTR to study the efficacy of ACP for the management of knee OA. These trials are summarized in Table 1. 

**Table 1:** Ongoing clinical trials registered on ClinicalTrials.gov, Clinical Trials Registry – India and Chinese Clinical Trial Register till January 4, 2024, evaluating the efficacy of autologous conditioned plasma (ACP) for the management of knee osteoarthritis.

Study Identifier	Biologic	Study Phase; Estimated Enrollment (N)	Primary Outcome Measure(s)	Recruitment Status	Country
NCT05765266	ACP Max vs. Depo-Medrol	Not applicable; N=45	Adverse Events – A comparison of the frequency and severity of all adverse events between the investigational group and the control group at 6 months; Adverse Events – Analysis of the frequency and severity of all adverse events for the investigational group at 12 months.	Not yet recruiting	USA
NCT03491761	ACP vs. hylauronic acid	Phase II; N=100	Cartilage Thickness on MRI - Evaluation of changes from baseline in central medial femorotibial compartment cartilage thickness measurements (via ordered value method) using quantitative T1 and cartilage compositional changes using T2 MRI at 6 and 12 months.	Unknown	USA
NCT06163573	ACP vs. N-TEC (autologous tissue engineered cartilage graft)	Phase II; N=75	Knee Injury and Osteoarthritis Outcome Score (KOOS) for pain - Mean change in Knee Injury and Osteoarthritis Outcome Score for pain from baseline to 24 months between groups Minimum score: 0 Maximum score :100 (higher score means no knee problems)	Not yet recruiting	Switzerland

Discussion

The present study evaluated the therapeutic potential of ACP for the management of knee OA. Clinical studies focusing on the effect of ACP on knee OA were included. Based on our search criteria and inclusion/exclusion criteria, five clinical studies fit the scope of our manuscript. Three ongoing clinical studies are registered on clinical trial protocol registries.

The early RCTs by Smith et al. [9] and Cerza et al. [10] demonstrated that administration of intraarticular ACP led to significant improvements in WOMAC score at 12 months and 24 weeks, respectively. In contrast, RCT by Cole et al. [8] reported no significant improvement in the WOMAC score. Although significant improvements were observed in VAS and IKDC scores up to 52 weeks follow-up, a decline in scores was reported after 24 weeks [8]. Moreover, more recent studies by Korpershoek et al. [6,7] evaluated the efficacy of ACP in knee OA patients in a real-world clinical practice and reported significant improvements in the KOOS score at 12 months, however this improvement did not reach MCID. This was attributed to the low concentration of platelets (<5x compared to baseline, i.e., whole blood concentration) in the injected ACP formulation [6-8]. This is in accordance with published studies that reported that a platelet concentration between 5x-7x compared to the baseline is needed to promote cell proliferation, mesenchymal stem cell recruitment and wound healing [11,12]. To address this shortcoming, Arthrex Inc. has designed a new Arthrex MaxTM PRP system to allow for obtaining higher platelet concentrations [13], which appears to be under investigation (NCT05765266; Table 1). In addition to the low platelet concentration, a potential reason for contrasting results between studies carried out in real-world clinical practice [6,7] versus RCTs [8-10] is the controlled conditions of the RCTs, i.e., in an RCT study subjects are selected with the aim of minimizing comorbidity and the protocol is designed to ensure highest providers and participants compliance [14]. Even though RCTs are considered the most reliable and the majority of the clinical guidelines are provided based on the outcomes deduced from them, it does not represent the divergent characteristics of real-world populations [14,15]. Thus, it is essential to integrate the real-world evidence with RCTs to complement it and to enhance the entirety of evidence-based medicine assessments [14,15]. 

## Conclusions

In conclusion, the aforementioned RCTs demonstrated the potential of ACP in alleviating symptoms associated with OA of the knee, however the MCID was not accomplished in real-world clinical settings. Thus, more adequately powered RCTs with longer follow-up as well as real-world post-market studies are warranted to establish long-term efficacy of ACP and justify its routine clinical use in patients suffering with knee OA.
